# P-1495. Immunogenicity and Safety of Recombinant High-Dose Compared to Egg-Based Standard-Dose Influenza Vaccine in Adults Living with Severe Obesity

**DOI:** 10.1093/ofid/ofaf695.1679

**Published:** 2026-01-11

**Authors:** Paul LOUBET, Sebastien Czernichow, Caroline Giboin, Ariane Sultan, Thomas Guimard, Emmanuel Disse, Marie-Pierre Tavolacci, Ines Ben Ghezala, Séverine Ledoux, Cécile Janssen, Helena Mosbah, Maeva Lefebvre, Arnaud De Luca, Liem Binh Luong Nguyen, Véronique Taillard, Julien Couster, Christian Duale, Corinne Dessaint, Claire Carette, Claire Rives-Lange, Sofia Zemouri, Florence Tubach, Odile Launay

**Affiliations:** CHU de Nîmes, Nimes, Languedoc-Roussillon, France; Assistance Publique Hôpitaux de Paris, Paris, Ile-de-France, France; Assistance Publique Hôpitaux de Paris, Paris, Ile-de-France, France; CHU de Montpellier, Montpellier, Languedoc-Roussillon, France; Centre Hospitalier Départemental Vendée, La Roche sur Yon, Poitou-Charentes, France; CH Lyon Sud, Lyon, Rhone-Alpes, France; Normandie Univ, UNIROUEN, U1073, CHU Rouen, and CIC-CRB 1404, F-76000 Rouen, France; F-CRIN, I REIVAC/COVIREIVAC, France, Rouen, Haute-Normandie, France; CHU Dijon, Dijon, Bourgogne, France; Assistance Publique Hôpitaux de Paris, Paris, Ile-de-France, France; CH Annecy Genevois, Annecy, Rhone-Alpes, France; CHU Poitiers, Poitiers, Poitou-Charentes, France; CHU de Nantes, Nantes, Pays de la Loire, France; CHU de Tours, Tours, Poitou-Charentes, France; Assistance Publique Hôpitaux de Paris, Paris, Ile-de-France, France; CHU de Nîmes, Nimes, Languedoc-Roussillon, France; CH de Boulogne, Boulogne, Nord-Pas-de-Calais, France; CHU Clermont-Ferrand, Clermont-Ferrand, Centre, France; Assistance Publique Hôpitaux de Paris, Paris, Ile-de-France, France; Assistance Publique Hôpitaux de Paris, Paris, Ile-de-France, France; Assistance Publique Hôpitaux de Paris, Paris, Ile-de-France, France; Assistance Publique Hôpitaux de Paris, Paris, Ile-de-France, France; Assistance Publique Hôpitaux de Paris, Paris, Ile-de-France, France; Université Paris Cité ; Inserm F-CRIN, I-REIVAC; Assistance Publique Hôpitaux de Paris, Paris, Ile-de-France, France

## Abstract

**Background:**

Individuals living with severe obesity face a high risk of severe flu and may have weakened immune responses to vaccination. The recombinant high-dose influenza vaccine (RIV) offers improved protection against influenza in adults aged 50 and older compared to an egg-based standard-dose inactivated influenza vaccine (SD). This open-label randomized trial assessed RIV's immune response and safety compared to SD among adults living with severe obesity.

**Table 1.**
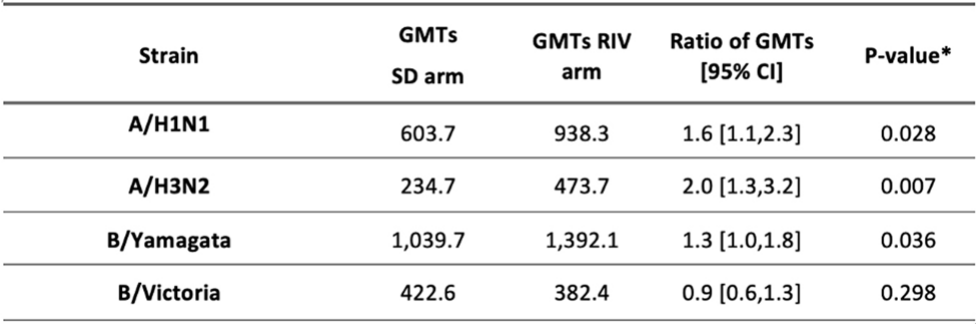
HAI geometric mean titers (GMTs) for each of the four strains on Day 28 and the ratio of GMTs (primary outcome) (N=206). * P-value of one-sided tests with Benjamin Hochberg correction to account for multiplicity

**Figure 1.**
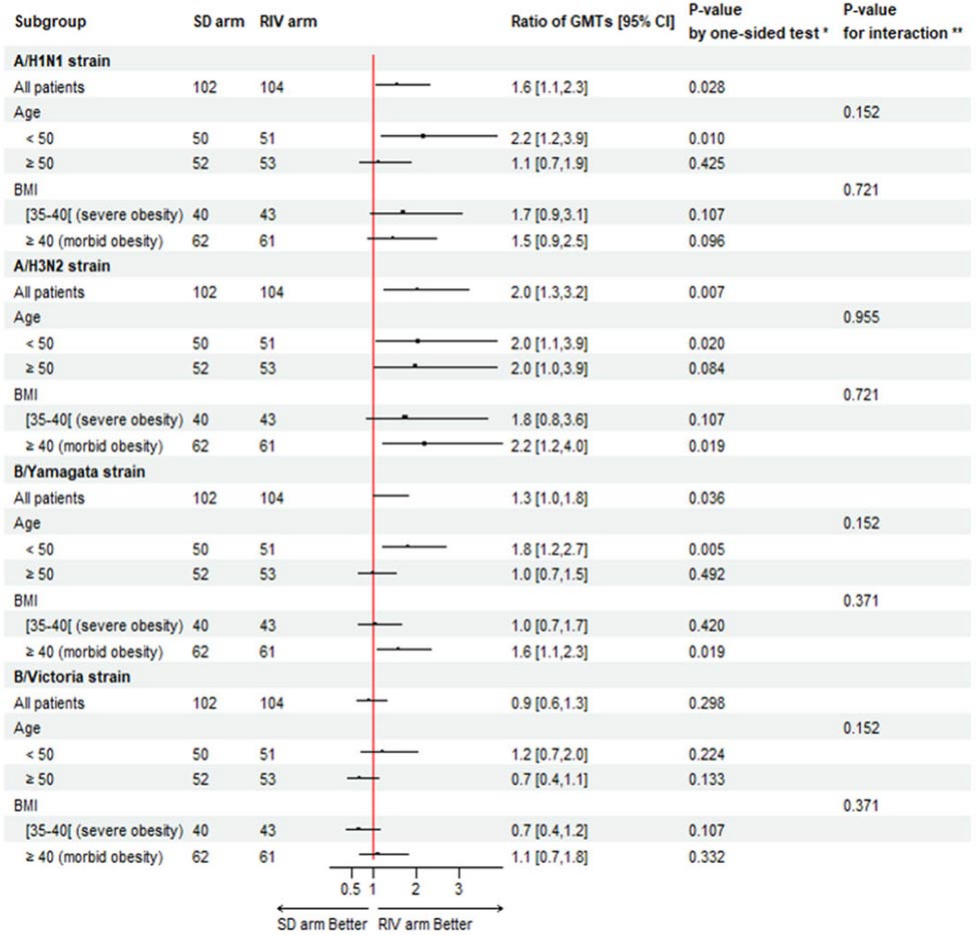
Subgroup analyses of the HAI geometric mean titers ratio at Day 28 (primary outcome). * P-value (one-sided tests with Benjamin Hochberg correction) ** P-value for interaction for subgroup analyses (two-sided tests with Benjamin Hochberg correction)

**Methods:**

Adult individuals with a body mass index (BMI) ≥ 35 kg/m² from 15 centers in France were randomized by minimization on center (1:1) to receive RIV or SD, stratified by age (< 50 or ≥50 years) and BMI ([35-40[ or ≥40] kg/m²). The primary outcome was the ratio (RIV/SD) of geometric mean hemagglutinin-inhibition (HAI) titers (GMTs) for each of the four strains 28 days after vaccination. Secondary outcomes included the HAI GMTs ratio at Day 180 post-vaccination, vaccine reactogenicity, and virologically confirmed influenza.

**Figure 2.**
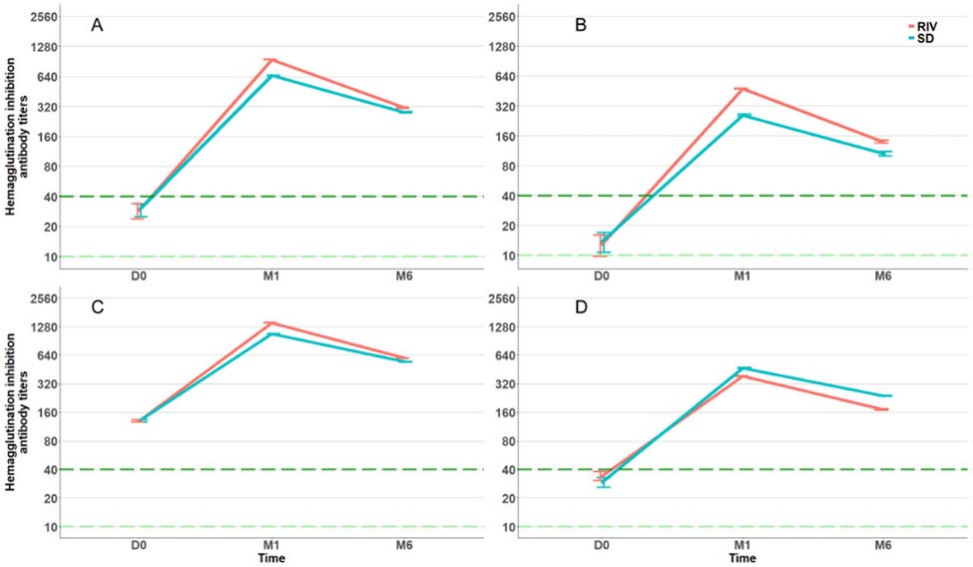
Evolution of HAI geometric mean titers over time for the strains A/H1N1 (A), A/H3N2 (B), B/Yamagata (C), and B/Victoria (D) by vaccination group.

**Results:**

A total of 206 participants were included (104 in the RIV group, 102 in the SD group) from November 2022 to March 2023. The median age was 50.0 years (interquartile range (IQR) 37.0-57.0) and the median BMI was 41.0 (IQR 38.1-45.7). At Day 28, the HAI GMTs ratio of RIV to SD was 1.6 (95% confidence interval (95% CI): 1.1-2.3) for A/H1N1, 2.0 (95% CI: 1.3-3.2) for A/H3N2, 1.3 (95% CI: 1.0-1.8) for B/Yamagata, and 0.9 (95% CI: 0.6-1.3) for B/Victoria. The HAI GMTs ratios were highest among younger participants (< 50 years) and those with a higher BMI (≥ 40.0 kg/m²). On Day 180, HAI GMT’s titers did not differ significantly. Reactogenicity was similar across all groups. There were no significant differences among the groups in influenza-like illness or RT-PCR-confirmed influenza.

**Conclusion:**

This is the first randomized trial to evaluate the humoral immune response of an enhanced influenza vaccine in a young, non-immunocompromised, at-risk population. The high-dose recombinant vaccine generated stronger humoral immune responses at Day 28 compared to an egg-based standard-dose vaccine in adults living with severe obesity, suggesting a potential additional benefit for the protection of these high-risk patients.

**Disclosures:**

Paul LOUBET, MD, PhD, Moderna: Advisor/Consultant|Moderna: Board Member|Pfizer: Advisor/Consultant|Sanofi: Advisor/Consultant|Sanofi: Board Member Odile Launay, MD, PhD, Merck: Advisor/Consultant|Merck: Board Member|Sanofi: Advisor/Consultant|Sanofi: Board Member

